# A haploid pseudo-chromosome genome assembly for a keystone sagebrush species of western North American rangelands

**DOI:** 10.1093/g3journal/jkac122

**Published:** 2022-05-14

**Authors:** Anthony E Melton, Andrew W Child, Richard S Beard, Carlos Dave C Dumaguit, Jennifer S Forbey, Matthew Germino, Marie-Anne de Graaff, Andrew Kliskey, Ilia J Leitch, Peggy Martinez, Stephen J Novak, Jaume Pellicer, Bryce A Richardson, Desiree Self, Marcelo Serpe, Sven Buerki

**Affiliations:** Department of Biological Sciences, Boise State University, Boise, ID 83725, USA; University of Idaho, Moscow, ID 83844, USA; Department of Biological Sciences, Boise State University, Boise, ID 83725, USA; Department of Biological Sciences, Boise State University, Boise, ID 83725, USA; Department of Biological Sciences, Boise State University, Boise, ID 83725, USA; Forest and Rangeland Ecosystem Science Center, United States Geological Survey, Boise, ID 83706, USA; Department of Biological Sciences, Boise State University, Boise, ID 83725, USA; University of Idaho, Moscow, ID 83844, USA; Royal Botanic Gardens, Richmond TW9 3AE, UK; Department of Biological Sciences, Boise State University, Boise, ID 83725, USA; Department of Biological Sciences, Boise State University, Boise, ID 83725, USA; Royal Botanic Gardens, Richmond TW9 3AE, UK; Institut Botànic de Barcelona (IBB, CSIC-Ajuntament de Barcelona), Barcelona 08038, Spain; Rocky Mountain Research Station, United States Forest Service, Moscow, ID 83843, USA; Department of Biological Sciences, Boise State University, Boise, ID 83725, USA; Department of Biological Sciences, Boise State University, Boise, ID 83725, USA; Department of Biological Sciences, Boise State University, Boise, ID 83725, USA

**Keywords:** *Artemisia tridentata*, keystone species, genomic resources

## Abstract

Increased ecological disturbances, species invasions, and climate change are creating severe conservation problems for several plant species that are widespread and foundational. Understanding the genetic diversity of these species and how it relates to adaptation to these stressors are necessary for guiding conservation and restoration efforts. This need is particularly acute for big sagebrush (*Artemisia tridentata*; Asteraceae), which was once the dominant shrub over 1,000,000 km^2^ in western North America but has since retracted by half and thus has become the target of one of the largest restoration seeding efforts globally. Here, we present the first reference-quality genome assembly for an ecologically important subspecies of big sagebrush (*A. tridentata* subsp. *tridentata*) based on short and long reads, as well as chromatin proximity ligation data analyzed using the HiRise pipeline. The final 4.2-Gb assembly consists of 5,492 scaffolds, with nine pseudo-chromosomal scaffolds (nine scaffolds comprising at least 90% of the assembled genome; *n *=* *9). The assembly contains an estimated 43,377 genes based on *ab initio* gene discovery and transcriptional data analyzed using the MAKER pipeline, with 91.37% of BUSCOs being completely assembled. The final assembly was highly repetitive, with repeat elements comprising 77.99% of the genome, making the *Artemisia tridentata* subsp. *tridentata* genome one of the most highly repetitive plant genomes to be sequenced and assembled. This genome assembly advances studies on plant adaptation to drought and heat stress and provides a valuable tool for future genomic research.

## Introduction

**Fig. 1.**
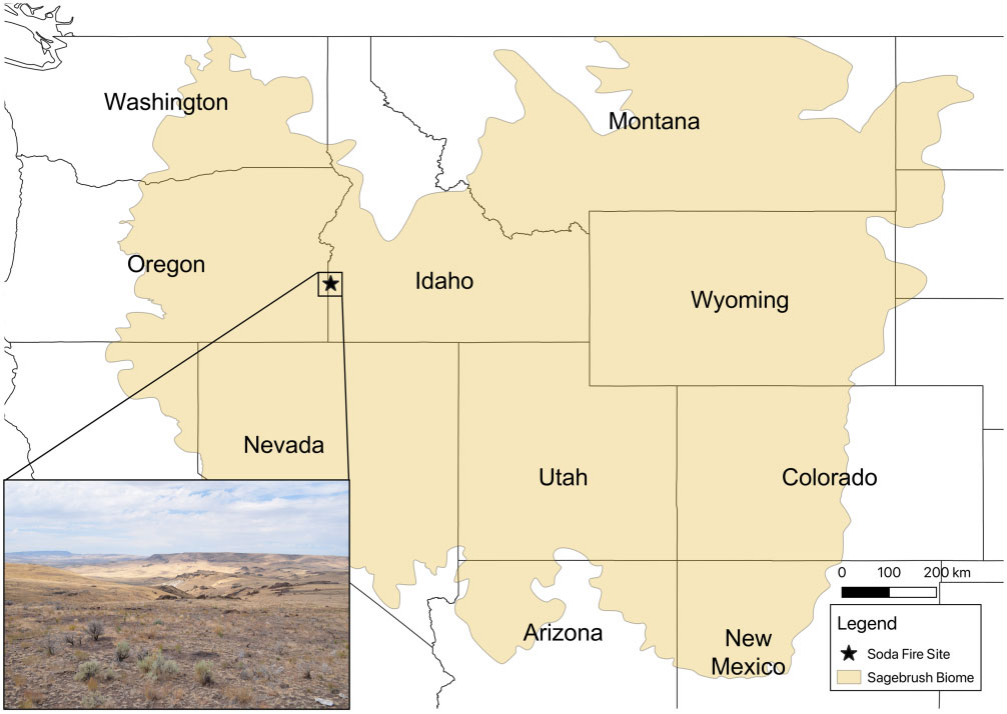
Map highlighting the sagebrush ecosystems and the site of collection of IDT3 within the Soda Fire site (burned in 2015) in Idaho, USA. Sagebrush ecosystems (also called the “Sagebrush Biome” per Rigge *et al.* 2020) currently cover an estimated range of 653,316 km^2^. The inset shows a landscape photo of the Soda Fire site.

Sagebrush ecosystems, comprising shrub and steppe dominated communities, are distributed across 14 western US states and two Canadian provinces (Fig. 1), and are dominated by endemic keystone sagebrush species of *Artemisia* L. subgenus *Tridentatae* (Rydb.) McArthur (McArthur *et al.* 1981; Garcia *et al.* 2011; Remington *et al.* 2021). These ecosystems are valued for livestock grazing, recreation, and wildlife habitat, but are pressured by altered climate, plant invasions, and wildfire, and thus intensive restoration efforts are underway (Baker 2006; Brabec *et al.* 2015; [Bibr jkac122-B46][Bibr jkac122-B46]). Sagebrush communities are recognized as some of the most imperiled suites of ecosystems worldwide with >350 species of plants and animals of conservation concern (Remington *et al.* 2021). Climatic niche models predict a 39% range reduction for the mid- to low-elevation sagebrush populations by mid-century due to rising temperatures (Still and Richardson 2015). This alarming prediction calls for research to prioritize the conservation and restoration of these taxa.

Big sagebrush (*Artemisia tridentata* Nutt.) shrublands once occupied ∼1,000,000 km^2^, but have been reduced by half due to the compound effects of climate change (Miller *et al.* 2012; Pilliod *et al.* 2017; O’Connor *et al.* 2020). Because big sagebrush does not re-sprout post-fire, ecosystem recovery only occurs via seedling recruitment (Wijayratne and Pyke 2012; Germino *et al.* 2018). Novel climatic conditions caused by climate change are creating conditions unsuitable for seedling recruitment therefore threatening the sustainability of sagebrush ecosystems (Pilliod *et al.* 2017).

Big sagebrush is a polyploid complex including three major subspecies—*A. tridentata* subsp. *tridentata*, *A. tridentata* subsp. *vaseyana*, and *A. tridentata* subsp. *wyomingensis* (hereafter referred to by subspecific epithets)—distributed across an environmental gradient with polyploids dominating the landscape (McArthur and Sanderson 1999). Subspecies *tridentata* and *vaseyana* exhibit both diploid (2*n *=* *2× = 18) and tetraploid (2*n *=* *4× = 32) cytotypes, whereas subspecies *wyomingensis* is only known as a polyploid (2*n *=* *4×, 2*n *=* *6× = 54) (McArthur and Sanderson 1999). Common garden experiments indicated that demographic phenotypes are under gene-by-environment control (Chaney *et al.* 2017). For example, a common garden experiment focusing on growth and fecundity rates was conducted to compare 2× *tridentata* and 4× *wyomingensis* performance across environments (Richardson *et al.* 2021). This study demonstrated that 2× *tridentata* outperformed 4× *wyomingensis*, even in environments dominated by polyploids (Richardson *et al.* 2021). The higher performance of 2× *tridentata* raised the question of how polyploids could be more prevalent in the landscape. A reference genome would provide genomic resources for future research aimed at increasing our understanding of observed phenotypes in common gardens, allow researchers to assess how big sagebrush populations have adapted to environmental changes, explain cytotype distributions, and provide a key resource to estimate the effect of climate change on its populations.

Here, we describe the first reference-quality genome assembly for 2× *A.tridentata* based on a clonally propagated individual line. A combination of short- and long-read and conformation capture sequencing technologies was used to assemble the 4.2 Gb haploid genome.

## Materials and methods

### Sample collection, in vitro tissue propagation, and biomass production

Previous studies have estimated the genome sizes of 2× *tridentata* and 4× *wyomingensis* to be 8.2 Gb/2C and 14.7 Gb/2C, respectively, suggesting an allopolyploid origin of the latter taxon (Garcia *et al.* 2008). This hypothesis was confirmed by phylogenetic analyses supporting polyphyly of 4× *wyomingensis*, and monophyly of 2× *tridentata* (Richardson *et al.* 2012). Previous research on a 2× *tridentata* draft genome has also suggested high genome complexity and levels of heterozygosity (Melton *et al.* 2021). Given the high heterozygosity, difference of genome sizes, and nonmonophyly of 4× *wyomingensis*, we focused on producing a reference genome for 2× *tridentata*. Due to the high genome complexity and outbred nature of the genome, an *in vitro* tissue propagation method was developed to provide sufficient biomass for genome sequencing and to allow for further experiments using plants of a single genotype that is shared with the reference genome (Barron *et al.* 2020).

Seeds used for tissue propagation came from a 2× *tridentata* mother plant known as IDT3 originating from the Soda Fire site (43.336 N, 116.964 W; Fig. 1) in the Northern Basin and Range ecoregion of Idaho, USA (Richardson *et al.* 2012). The taxonomy and ploidy level of the mother plant were confirmed using morphological features coupled with phylogenetic analyses and flow cytometry (Richardson *et al.* 2012; Chaney *et al.* 2017). An *in vitro* method of propagation for 2× *tridentata* developed by Barron *et al.* (2020) was used to produce biomass for IDT3 “G1_b2” by harvesting leaf tissue (average of 1.7 g per plantlet) from 15-week-old plantlets. The ploidy level and genome size of “G1_b2” were confirmed using flow cytometry (see below). Genome complexity and level of heterozygosity were estimated using a *k*-mer approach as implemented in GenomeScope (Vurture *et al.* 2017) using Illumina short-reads (see below). Based on these results, we estimated that 120 g of fresh leaf biomass was required to extract sufficient high quality and high-molecular weight DNA (fragment size greater than 50 kb) using a CTAB DNA extraction protocol for genome sequencing to sequence a genome at 100× coverage, *de novo* genome assembly, and scaffolding using OmniC proximity-ligation sequencing and the HiRise pipeline. This amount of tissue corresponded to 71 “G1_b2” plantlets. It took seven months to generate the necessary biomass while also maintaining the individual line in culture at Boise State University. Prior to biomass harvesting, plantlets were dark-treated for 48 h. The biomass was then flash frozen using liquid nitrogen and shipped overnight on dry ice to DovetailGenomics (Scotts Valley, California, USA) where DNA and RNA extractions were conducted (see below). For RNA extractions, 1 g of root biomass was also provided to complement the leaf biomass, both of which were used for genome annotation (see below).

### Flow cytometry and genome complexity analysis

Flow cytometry was performed using methods outlined in Garcia *et al.* (2008) and Pellicer and Leitch (2014). Briefly, leaf material of G1_b2 was cochopped with the calibration standard *Petunia hybrida* Vilm. “PxPc6” (2C = 2.85 pg) in General Purpose Buffer (Loureiro *et al.* 2007) and stained using the base-independent fluorochrome propidium iodide. The samples were analyzed at Boise State University using a BD Accuri C6 Flow Cytometer with approximately 10,000 events (i.e. DNA fluorescence for approximately 10,000 nuclei) being recorded. Genome size was calculated per equation in (Pellicer and Leitch 2014).

The QIAGEN DNeasy Plant mini kit (Hilden, Germany; catalogue # 69204) was used to extract genomic DNA for short-read sequencing using 20 mg of dried leaf tissue per manufacturer protocol. To assess genome size and complexity, whole-genome sequencing (2 × 150 bp; genome coverage ∼160× read depth) was conducted on five lanes of Illumina HiSeq X (San Diego, CA, USA) by GeneWiz (New Jersey, NJ, USA). Raw read data were cleaned using Trimmomatic v.0.36 (Bolger *et al.* 2014). A subset of 1.05 × 10^11^ cleaned reads were then used to generate *k*-mers (*k *=* *21) with KMCTools V3.1.1 (Kokot *et al.* 2017) for assessing genome size and complexity with the online GenomeScope portal (Vurture *et al.* 2017) and the R package “Smudgeplot” V0.2.4 (Ranallo-Benavidez *et al.* 2020). The lower and upper thresholds for *k*-mer coverage were 18 and 3,700, respectively, per the cutoff function from the Smudgpelot python script for the Smudgeplot analysis, limiting the inclusion of sequencing error (lower limit) and homozygous duplicate *k*-mers (upper limit).

### PacBio and Omni-C sequence data generation

PacBio long-read and OmniC proximity-ligation sequence data production for the “G1_b2” genome assembly were performed as follows: (1) extract high-molecular weight DNA from 120 g of leaf biomass, (2) conduct whole-genome sequencing using PacBio long-read technology to produce ∼100× raw data coverage, and (3) prepare and sequence Dovetail Omni-C proximity-ligation libraries to further scaffold the de novo genome. These analyses were performed by DovetailGenomics.

High-molecular weight DNA was extracted using the CTAB method (Doyle and Doyle, 1987). DNA samples were quantified using Qubit 2.0 Fluorometer (Life Technologies, Carlsbad, CA, USA). A total of five PacBio SMRTbell libraries (∼20 kb) for PacBio Sequel were constructed using a SMRTbell Express Template Prep Kit 2.0 (PacBio, Menlo Park, CA, USA) following the manufacturer-recommended protocol. Each library was bound to polymerase using the Sequel II Binding Kit 2.0 (PacBio) and loaded onto the PacBio Sequel II instrument. Each library was sequenced individually on PacBio Sequel II 8M SMRT cells for a total of five sequencing runs.

Three Dovetail Omni-C libraries were prepared for proximity-ligation analysis. To prepare these libraries, chromatin was fixed with formaldehyde in the nucleus and then extracted using the QIAGEN blood and cell culture DNA mini kit (Hilden, Germany; catalogue # 13343). Fixed chromatin was digested with DNAse I, chromatin ends were repaired and ligated to a biotinylated bridge adapter followed by proximity ligation of adapter containing ends. After proximity ligation, crosslinks were reversed and the DNA was purified. Purified DNA was treated to remove biotin that was not internal to ligated fragments. Sequencing libraries were generated using NEBNext Ultra enzymes and Illumina-compatible adapters (New England BioLabs, Hitchin, UK). Biotin-containing fragments were isolated using streptavidin beads before PCR enrichment of each library. The libraries were sequenced on an Illumina HiSeq X platform at approximately 30× sequence coverage.

### PacBio long-read *de novo* assembly and validation

A *de novo* assembly of the resulting PacBio continuous long reads was performed using WTDBG2 v2.5 (Ruan and Li 2020) with the following parameters: genome size 5.0 Gb, minimum read length 20,000, and minimum alignment length of 8,192 bp. Additionally, realignment was enabled with the -R option and read type was set with the option -x sq. To identify potential contaminants, the *de novo* assembly was assessed using a BLAST (Altschul *et al.* 1990) search against a database of nucleotide sequences from NCBI. BLAST results of the de novo assembly against the nucleotide database were assessed using blobtools v1.1.1 (Laetsch *et al.* 2020). Scaffolds identified as possible contamination using BLAST and blobtools were then removed from the assembly. Finally, purge_dups v1.2.3 (Guan *et al.* 2020) was used to remove haplotigs and highly overlapping contigs.

### Pseudomolecule construction with HiRise

**Fig. 2.**
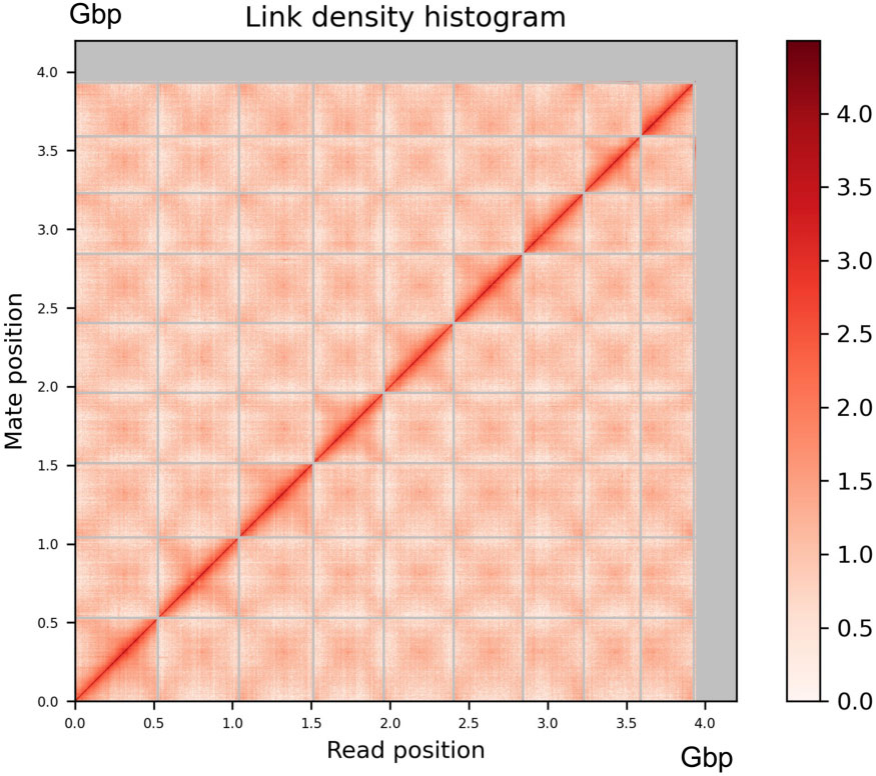
Linkage–density histogram for the HiRise assembly generated by Dovetail Genomics. The axes represent the mapping positions along the genome assembly of the first (*x*-axis) and second (*y*-axis) read in the read pair, grouped into bins. The color of each square represents the number of reads within a given bin, with darker colors indicating more reads being mapped within the given bin. Vertical and horizontal lines have been added to delimit the scaffolds (smaller scaffolds are not visible in the plot due to scale and are represented by the large gray lines at the upper limits of the *X*- and *Y*-axes). *X* and *Y*-axes represent the position within the genome assembly in Gb, with pseudo-chromosomal scaffolds ordered largest to smallest.

The de novo assembly and Dovetail Omni-C library reads were used as input data for HiRise, a software pipeline designed specifically for using proximity ligation data to scaffold genome assemblies (Putnam *et al.* 2016). Dovetail Omni-C library sequences were aligned to the draft input assembly using bwa (Li and Durbin 2009). The separations of Dovetail Omni-C read pairs mapped within draft scaffolds were analyzed by HiRise to produce a likelihood model for genomic distance between read pairs, and the model was used to identify and break putative misjoins, to score prospective joins, and make joins above a threshold (Fig. 2). The final HiRise assembly was assessed for completeness using the eukaryota_odb10 database in BUSCO V4.0.5 (Benchmarking Universal Single-Copy Orthologs; Simão *et al.* 2015).

### Genome annotation

The genome was annotated for both noncoding repetitive DNA and for functional, coding genes. Preliminary functional annotation was performed using *ab initio* gene discovery and transcriptional data.

#### RNA sequencing

Illumina short-read RNA-Seq was performed to support annotation of the genome assembly. Total RNA extractions for leaf and root tissues were performed using the QIAGEN RNeasy Plus Kit following manufacturer protocols (Hilden, Germany). Total RNA was quantified using Qubit RNA Assay (Thermo Fisher Scientific, Waltham, MA, USA) and TapeStation 4200 (Agilent, Santa Clara, CA, USA). Prior to library prep, DNase treatment was performed followed by AMPure bead clean up (Beckman Coulter Life Sciences, Indianapolis, IN, USA) and QIAGEN FastSelect HMR rRNA depletion (Hilden, Germany). Library preparation was performed with the NEBNext Ultra II RNA Library Prep Kit following manufacturer protocols. These libraries were then sequenced on an Illumina NovaSeq6000 instrument in the 2 × 150 bp configuration.

#### Repeat identification

Repeat families found in the genome assemblies of 2× *tridentata* were identified *de novo* and classified using the software package RepeatModeler v.2.0.1 (Flynn *et al.* 2020). RepeatModeler depends on the programs RECON v.1.08 (Bao and Eddy 2002) and RepeatScout v.1.0.6 (Price *et al.* 2005) for the *de novo* identification of repeats within the genome. The custom repeat library obtained from RepeatModeler was used to discover, identify, and mask the repeats in the assembly file using RepeatMasker v.4.1.0 (Smit *et al*. 2013 ).

#### Functional annotation

Coding sequences from *Cynara cardunculus* L., *Erigeron canadensis* L., *Helianthus annuus* L., *Lactuca sativa* L., and *Mikania micrantha* Kunth. were used to train the initial *ab initio* gene discovery model for 2× *tridentata* using the AUGUSTUS software v.2.5.5 (Stanke *et al.* 2004). Six rounds of prediction optimization were done with the software package provided by AUGUSTUS. The same coding sequences were also used to train a separate *ab initio* gene discovery model for 2× *tridentata* using SNAP v.2006-07-28 (Korf 2004). RNA-Seq reads were mapped onto the genome using the STAR aligner software v.2.7 (Dobin *et al.* 2013) and intron hints (i.e. extrinsic evidence about the location and structure of genes) generated with the *bam2hints* tools within the AUGUSTUS software. MAKER (Cantarel *et al.* 2008), SNAP (Korf 2004), and AUGUSTUS (Stanke *et al.* 2004) (with intron–exon boundary hints provided from RNA-Seq) were then used to predict genes in the repeat-masked reference genome. To help guide the prediction process, Swiss-Prot peptide sequences from the UniProt (UniProt Consortium 2019) database were downloaded and used in conjunction with the protein sequences from *C. cardunculus*, *E. canadensis*, *H. annuus*, *L. sativa*, and *M. micrantha* to generate peptide evidence in the MAKER pipeline. Only genes that were predicted by both SNAP and AUGUSTUS were retained in the final gene sets. To help assess the quality of the gene prediction, Annotation Edit Distance scores (Eilbeck *et al.* 2009), a metric to quantify the amount of change between individual annotations, were generated for each of the predicted genes as part of the MAKER pipeline. Genes were further characterized for their putative function by performing a BLAST search of the peptide sequences against the UniProt database. tRNA were predicted using the software tRNAscan-SE v.2.05 (Chan *et al.* 2021). Finally, to meet NCBI genome submission standards, seven scaffolds of 200 bases or less and one scaffold comprising a mitochondrial genome fragment were removed from the annotated HiRise assembly.

## Results and discussion

### Validation of genome assembly and annotation

**Fig. 3.**
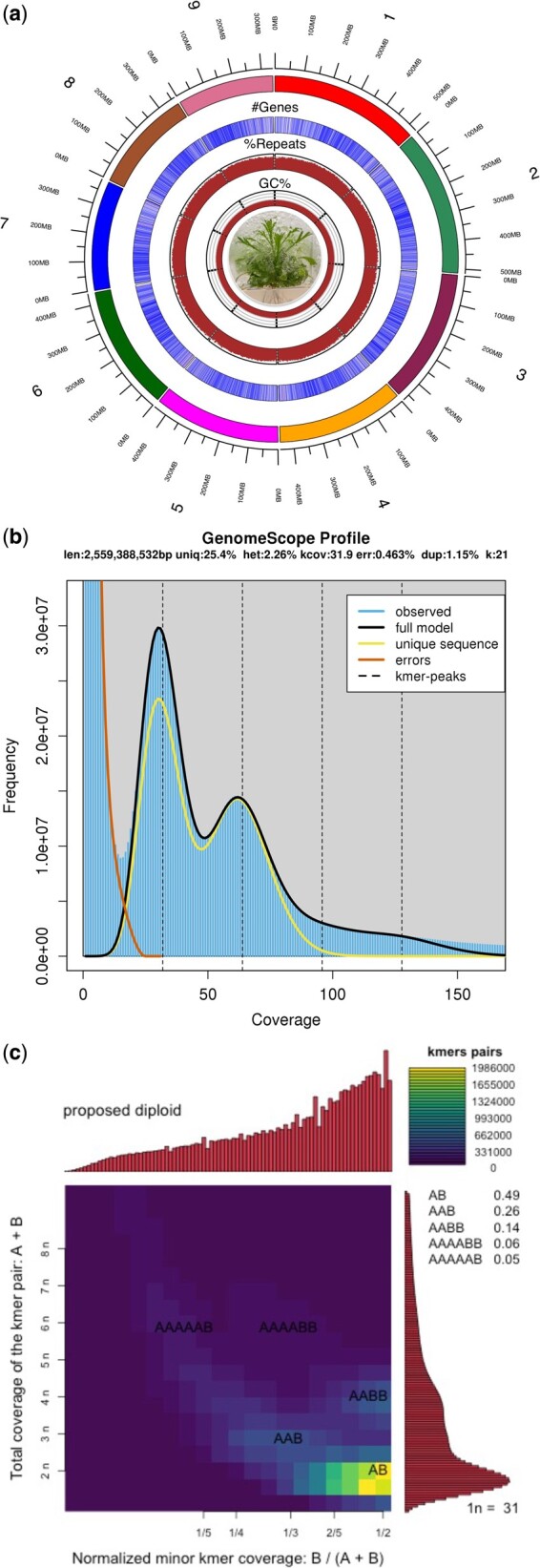
Density plot of *k*-mer analysis in GenomeScope and genome map showing GC content (%), % repeat per 1 million nucleotides, number of genes per 1 million nucleotides, and the size of the scaffold for the nine pseudo-chromosomal scaffolds. Subset (a) shows the genome feature mapping for the nine pseudo-chromosomal scaffolds, subset (b) shows GenomeScope results, and subset (c) shows the Smudgeplot results. GenomeScope summary statistics, including heterozygosity rate (listed as “het”), are listed at the top of plot (b). Two primary *k-*mer peaks are present, indicating that the genome is diploid. The Smudgeplot shows the frequency of *k-*mer pairs within the genome, with darker colors indicating the group is less frequent and bright yellow indicating the group is more frequent. When visualized, the plot shows distinct “smudges” representing each *k*-mer pair with the greatest of density of *k*-mers relating to the ploidy level of the genome (e.g. the diploid *A. tridentata* genome has the brightest “smudge” for the diploid *AB k*-mer pair).

The final processed 2× *tridentata* genome assembly comprises 5,492 scaffolds, nine of which are pseudo-chromosomes (L90 = 9 = *n*), and 4,198,553,833 bases (4.20 Gb; Fig. 3a). The pseudo-chromosome scaffolds range from 0.528 to 0.338 Gb in length (Fig. 3a and Table 1). Flow cytometry on the IDT3 “G1_b2” sample estimated the genome size to be 4.19 Gb/1C, which is in line with previous estimates of the 2× *tridentata* genome sizes (i.e. 4.1 Gb/1C per Garcia *et al.* 2008). The GenomeScope and Smudgeplot analyses further confirmed the genome to be diploid, with two distinct *k-*mer peaks in the GenomeScope plot and greatest density of *k*-mers in the diploid *AB* “smudge” in the Smudgeplot, and revealed high levels of genome complexity, with evidence of past hybridization, polyploidization-to-diploidization events, and high levels of out-crossing (Fig. 3, b and c). These results are consistent with previous studies that found evidence of past polyploidy and hybridization events within *Artemisia* (e.g. Garcia *et al.* 2008; Barron *et al.* 2020).

**Table 1.** Summary statistics for the 9 pseudo-chromosomal scaffolds within the IDT3 “G1_b2” genome assembly.

Scaffold	Length in Gb (% of assembly)	Protein coding genes	Total gene length in Gb (% of assembly)	Repeat occurrences	Repeat length total in Gb (% of assembly)
1	0.528 (12.58)	5,869	0.018 (3.49)	709,220	0.444 (84.00)
2	0.514 (12.23)	5,153	0.015 (2.99)	682,886	0.443 (86.21)
3	0.472 (11.24)	4,781	0.015 (3.15)	624,680	0.406 (86.04)
4	0.446 (10.62)	4,707	0.015 (3.33)	591,412	0.378 (84.73)
5	0.445 (10.59)	4,951	0.017 (3.73)	591,818	0.371 (83.43)
6	0.439 (10.46)	4,358	0.013 (3.04)	580,217	0.379 (86.38)
7	0.385 (9.18)	4,096	0.013 (3.30)	513,867	0.330 (85.52)
8	0.361 (8.61)	3,520	0.011 (3.03)	480,240	0.311 (86.11)
9	0.338 (8.06)	3,430	0.011 (3.11)	446,444	0.295 (87.12)
Total	3.929 (93.58)	40,865	0.128 (3.25)	5,220,784	3.356464852 (85.43)

Repeat identification analysis revealed that the 2× *tridentata* genome is highly repetitive. A total of 77.99% of the genome consisted of repetitive sequences (Fig. 3a), with the largest class being Class I Transposable Elements (TE; 36.20%), with Class II TEs being the second most common repeat (2.19%) (Tables 1 and 2). Low complexity and simple repeats comprise 0.10% and 0.82% of the genome assembly, respectively. A total of 85.43% of the pseudo-chromosome scaffold sequence was found to be repetitive, with an average of 85.50% for each pseudo-chromosome (Table 1). This level of repetitive DNA sequence is high since the average repetitive DNA content for plant genomes is 57%, with relatively few plant genomes containing >75% repetitive sequence (Michael and Jackson 2013; Michael 2014), making the 2× *tridentata* genome one of the most highly repetitive plant genomes sequenced.

**Table 2.** Summary statistics for the *de novo* and HiRise genome assembly outputs.

	De novo assembly	HiRise assembly
Total length (bp)	4,197,847,053	4,198,560,453
N50	965,994	444,777,032
L50	1,188	5
N90	246,927	338,336,202
L90	4,521	9
Largest scaffold (bp)	10,654,198	528,210,163
Number of scaffolds	12,613	5,500
Number of scaffolds >1 kb	12,577	5,464
Number of gaps	1,859	8,993
Number of *N*'s/100 kb	1	18
Complete BUSCOs (C)	232 (90.98%)	233 (91.37%)
Complete and single-copy BUSCOs (S)	175 (68.63%)	188 (73.73%)
Complete and duplicated BUSCOs (D)	57	45
Fragmented BUSCOs (F)	2	5
Missing BUSCOs (M)	21	17
Total BUSCO groups searched	255	255

The final assembly, with scaffolds <200 bases in length and 1 mitochondrial fragment removed, totaled 4,198,553,833 bases and comprised 5,492 scaffolds.

Benchmarking Universal Single-Copy Orthologs (BUSCO) analysis recovered 91.37% (233 of 255) of single-copy BUSCOs from the HiRise assembly. A total of 1.2% of BUSCOs were found to be duplicated. Only 3.1% of BUSCOs were fragmented and 9.0% were missing (Table 2). This result indicated a high level of completeness in the genome assembly and that the genome was sufficiently assembled for annotation. Using *ab initio* gene discovery and transcriptomic evidence, a total of 43,377 genes were identified, with coding regions comprising 0.59 Gb. Of the 43,477 genes identified, 40,865 were located on the pseudo-chromosome scaffolds, with each scaffold containing an average of 4,541 genes (Table 1). The average length of these genes was 1,358 bp. A total of 8,759 genes were found to comprise a single exon.

### Genome complexity and evidence of past polyploidization

The GenomeScope analysis showed that the 2× *tridentata* genome is a highly heterozygous genome, with an estimated level of heterozygosity of 2.26% (listed as het: 2.26% in Fig. 3b). This is relatively high when compared to other plants, such as *Arabidopsis thaliana* (1.04%), and slightly less than the highly heterozygous *Vitis vinifera* genome (2.6%; Jaillon *etal*. 2007). The Smudgeplot analysis (Fig. 3c) revealed that while diploid (as shown by the highest *k*-mer coverage being that of 2*n k*-mers), there are varying levels of coverage depth for the different *k*-mer pairs, indicating a complex evolutionary history including prior hybridization and polyploidization events. The diploid *AB k*-mer pairs were most prominent (49% of *k*-mers), the *AAB* and *AABB k*-mer pairs were the next most common at 26% and 14% of *k*-mers, respectively (Fig. 3c). Greater *AABB k*-mer pairs than *AAAB k*-mer pairs would be indicative of past allopolyploidization via hybridization and genome doubling, with equivalent contributions of the *A* and *B* parental genomes (Ranallo-Benavidez *et al.* 2020). The higher proportion of *AAB* would suggest backcrossing with the diploid parental *A* genome after the allopolyploidization event. While our results indicated “G1_b2” is a diploid, the 2× *tridentata* genome demonstrated evidence of past polyploidization followed by chromosomal rearrangements leading to diploidy (i.e. diploidization; Dodsworth *et al.* 2016). Such a process has been advocated to be one of the main drivers of the evolutionary success of flowering plants and further studying it in sagebrush could shed light into the mechanisms of adaptations leading to the diversification of this lineage in the sagebrush steppes (Dodsworth *et al.* 2016).

### Comparing the *A. tridentata* and *A. annua* genome assemblies

*Artemisia annua* L., commonly known as sweet wormwood, is the only other species of *Artemisia* to have its genome sequenced (Shen *et al.* 2018). The *A. annua* genome assembly represents a fairly high-quality draft assembly, containing 39,579 scaffolds (Shen *et al.* 2018). While the divergence of the clades containing *A. annua* and *A. tridentata* occurred ∼10.8 MYA (Sanz *et al.* 2011), these species maintain a conserved ploidy level, with the base karyotype number for each species comprising nine chromosomes (2*n *=* *2× = 18; McArthur *et al.* 1981). While these species contain the same number of chromosomes, there are distinct differences in their genomes. The genome size for *A. tridentata*, and other members of the North American *Tridentatae* subgenus (Garcia *et al.* 2008; Pellicer *et al.* 2010), is nearly 2.5 times the size of the *A. annua* genome (4.20 Gb/1C vs. 1.74 Gb/1C). The current genome assembly of *A. annua* has been found to contain more genes (63,226 genes; Shen *et al.* 2018) than identified here in the genome assembly and annotation for *A. tridentata* (43,377 genes). This difference in gene content may be partially explained by incomplete annotation of paralogues, particularly tandem paralogues whose annotations can be merged into one (Campbell *et al.* 2014). Tandem paralogues have been previously identified in a draft assembly of the *A. tridentata* genome, in which two tandem Aquaporin paralogues were found on one scaffold (Melton *et al.* 2021). Future comparative genomic and transcriptomic analyses will need to be performed to ascertain whether gene content is higher in the *A. annua* genome than in the *A. tridentata* genome or if incorrect annotation of tandem paralogs in the *A. tridentata* genome has led to an underestimation of gene content.

The genome of *A. tridentata* is far more heterozygous (2.26% vs. 1.0–1.5%) and repetitive (77.99% vs. 61.57%) than the *A. annua* genome. These aspects of the *A. tridentata* genome are likely the result of a polyploidization, likely due to an allopolyploidization event, early within the divergence of subgenus *Tridentatae* followed by diploidization (Garcia *et al.* 2008; Pellicer *et al.* 2010), also supported by high proportion of *AB k*-mer pair, with lower proportions of *AAB* and *AABB k*-mer pairs, and greatest density of *k*-mers in the diploid *AB* “smudge” presented in the Smudgeplot results here (Fig. 3c). Differences in the assembly levels may also contribute to the perceived differences in repetitiveness, as repetitive genome sequences are difficult to quantify in more fragmented genomes.

### Applications of the sagebrush reference genome

The 2× *tridentata* genome sequence data will serve as a valuable resource for a broad range of researchers. This species has been used to study abiotic stress responses using physiological and eco-physiological methods for decades (DePuit and Caldwell 2017; Richards and Caldwell 1987; Kolb and Sperry 1999; Ryel *et al.* 2004; Germino 2012; Copeland *et al.* 2022). This genome will allow for greater connectivity between field-based and ecophysiological research and genomic research, which aims to elucidate genome-to-phenome and stress-response pathways. *Artemisia tridentata* also belongs to the ecologically and economically important Asteraceae family comprising 10% of angiosperm diversity (Mandel *et al.* 2019), allowing this genome to serve as an important contribution to our understanding of Asteraceae evolution. Currently, 24 Asteraceae genomes are available through NCBI and this genome fills a taxonomic and phylogenetic gap in Asteroideae (Supplementary Table 1). For these genome assemblies, the average size is 1.59 Gb (standard deviation = ± 1.06 Gb), much smaller than the 4.20 Gb assembly for *Artemisia tridentata.* This new Asteraceae genome assembly and the variation in genome size within the family allow for further research into the processes that shape genome size. *Artemisia* is also amongst the largest genera of Asteraceae with species of agricultural, horticultural, medicinal, and pharmaceutical importance (Bora and Sharma 2011; Pellicer *et al.* 2011, 2018). The antimalarial agent artemisinin was detected in multiple species of *Artemisia*, including *Artemisia tridentata*, demonstrating the potential usage of genomic data for studying the evolution of biochemical pathways relevant to potential drug discovery (Pellicer *et al.* 2018).

## Data availability

Supplementary Table 2 lists all sequence data generated in this project. All sequence data for this project are available from the National Center for Biotechnology Information (NCBI) under BioProject accession number PRJNA795150 and BioSample accession number SAMN24662005. The Whole Genome Shotgun project has been deposited at DDBJ/ENA/GenBank under the accession JAKJXK000000000. All raw sequence files are available from the NCBI SRA database (PacBio long read data SRR17863255 Omni-C proximity-ligation data SRR17863200, SRR17870744 and SRR17870745; Illumina HiSeq short read data SRR17870775 and SRR17863368; RNASeq paired end reads from leaf tissue SRR17779362; RNASeq paired end reads from root tissue SRR17779361). Genome annotation results and supporting data files are openly available via the G3 figshare repository at https://doi.org/10.25387/g3.19651260.

All software used in this work is in the public domain, with parameters being clearly described in Materials and methods. If parameters were not detailed for a software, default parameters were used as suggested by the developer.

Supplemental material is available at *G3* online.

## Supplementary Material

jkac122_Supplemental_Table_1

jkac122_Supplemental_Table_1

jkac122_Supplemental_Figure_1
