# Supplementary material for: A haploid pseudo-chromosome genome assembly for a keystone sagebrush species of western North American rangelands
Source: G3 (Bethesda). 2022 May 14;12(7):jkac122. doi: 10.1093/g3journal/jkac122 (PMC9258541; doi:10.1093/g3journal/jkac122)
Supplement: jkac122_Supplemental_Figure_1 [file jkac122_supplemental_figure_1.docx]

Supplemental Figure 1. Diploid *tridentata* plantlets growing *in vitro* and *ex vitro*. IDT3 “G1_b2” was propagated using *in vitro* methods (Barron et al, 2020) to generate sufficient biomass for high molecular weight DNA extraction and whole genome sequencing. This line is maintained using *in vitro* (A) and *ex vitro* (B) methods at Boise State University.
